# 

**Published:** 1908-08-29

**Authors:** 


### CONTENTS.

| | PAGE |
|---|---|
| **The Health of the Army** ... | 563 |
| **The Burden of General Practice** ... | 564 |
| **Annotations—** Cancer and Tubercle—Doctors and their Fees—Maxims of an Old Physician ... | 565 |
| **Medical Opinion and Movement** ... | 566 |
| **Hospital Clinics—** The Results of Operations for Cancer of the Breast. By T. Crisp English, B.S., F.R.C.S. ... | 569 |
| **Special Article—** Points in the Treatment of Fistula-in-Ano ... | 571 |
| **Medicine—** Myocarditis and Myocardial Disease—IV. ... | 573 |
| The X-rays in Exophthalmic Goitre ... | 574 |
| **Surgery—** Œsophageal Obstruction.—IV.... | 575 |
| **Laryngology and Rhinology—** Enlargement of the Tonsils—I. ... | 576 |
| **Anæsthetics—** The Treatment of an Impending Calamity... | 577 |
| **Notes on Treatment—** Warts and the X-rays ... | 578 |
| **Ophthalmology—** The Treatment of Trachoma ... | 579 |
| **Resident Medical Officers' Department—** On History in Acute Cases ... | 580 |
| **The General Practitioner's Column—** Practical Hints on the Management of Normal and Complicated Breech Cases ... | 581 |
| **Therapeutics—** Polygonum Cuspidatum as a Laxative ... | 582 |
| **Hospital Administration—** The London Fever Hospital ... | 583 |
| **News and Coming Events** ... | 585 |
| **New Appliances and Things Medical** ... | 586 |
| **Nursing Administration—** The Domestic Staff—II. ... | 587 |
| **The Graduates' Medical Schools** ... | 588 |
| **Editor's Letter-Box** ... | 588 |

